# 

**Published:** 1893-12

**Authors:** 


### CONTENTS FOR DECEMBER, 1893.

ORIGINAL COMMUNICATIONS. PAGE.

Torsion and the Homologous Ligation of Divided Arteries. By Thomas H. Manley, M. D. .... 257
What Kills Patients After Laparatomy—Anesthetic, Nephritis, or Infection? By F. Byron Robinson, Chicago, Ill. .... 272
Laryngeal Tuberculosis—A Typical Case, with Illustration. By Horace Clark, M. D. .... 275
Tricuspid Insufficiency. By Frank J. Thornbury, M. D. .... 278

CLINICAL REPORTS.

Clinical Memoranda from the Surgical Clinic at the Sisters' of Charity Hospital. By Herman Mynter, M. D. .... 281
A Case of Angio-Neurotic Edema, Showing Remarkable Heredity. By William C. Fritz, M. D. .... 286

SELECTIONS.

The Evils of Substitution .... 287
The Indications for Trional .... 290
Surgical Treatment of Urinary Incontinence in the Woman .... 291

EDITORIAL.

The Southern Surgical and Gynecological Association .... 292
Topics of the Month .... 296

Personal .... 300
Obituary .... 301
Society Meetings .... 302
Academy of Medicine Notes .... 303
Book Reviews .... 303
Books Received .... 317
Literary and Miscellany .... 319

